# Perceived benefits and risks: A survey data set towards Wolbachia-infected *Aedes* Mosquitoes in Klang Valley

**DOI:** 10.1016/j.dib.2020.106262

**Published:** 2020-09-02

**Authors:** Ahmad Firdhaus Arham, Latifah Amin, Muhammad Adzran Che Mustapa, Zurina Mahadi, Ahmad Fadhly Arham, Mashitoh Yaacob, Maznah Ibrahim, Nor Sabrena Norizan

**Affiliations:** a Pusat Citra Universiti, Universiti Kebangsaan, Malaysia; b Faculty of Business and Management, Universiti Teknologi Mara (UiTM), Melaka, Malaysia

**Keywords:** Perceived benefits, Perceived risks, Attitude, Wolbachia-infected *Aedes* mosquitoes, Malaysia

## Abstract

Perceived Benefits and Risks: A survey data set towards Wolbachia-infected *Aedes* Mosquitoes in the Klang Valley, Malaysia.

Introduction: The paper presents data collected using measures of perceived benefits, perceived risks, trust in key players, attitude towards nature versus material, attitude towards technology, religiosity, and attitude towards the Wolbachia-infected *Aedes* mosquitoes (WiAM) technique. The validated questionnaires were used to randomly survey targeted stakeholders in the Klang Valley, Malaysia, who had been asked to voluntarily participate in face-to-face interviews. Completed questionnaires were received from 399 respondents (adults above 18 years old) and comprised two stakeholder groups: scientists (*n* = 202), and the public (*n* = 197). The detailed findings serve numerous opportunities to examine the social acceptance of Wolbachia-infected *Aedes* mosquitoes, to ensure the development of policy and action plans, and to encourage further study by other researchers interested in the measures and data presented.

## Specifications Table

**Table:** 

Subject	Infectious Diseases; Environmental Health.
Specific subject area	Dengue Control and Prevention Technique; Social Acceptance; Attitude and Behaviour; Perceived Benefits; Perceived Risks; Trust in Key Players; Attitude towards Nature vs Material; Attitude towards Technology; Religiosity.
Type of data	Table, Figure, Excel file.
How data were acquired	Survey through a structured questionnaire.
Data format	The data is in raw format and has been analysed with descriptive and statistical details. The data file has been clean uploaded.
Parameters for data collection	Samples were made up of scientists and the public. The sample was randomly selected from the dengue hot spot area identified as having the highest incidence of dengue fever cases as reported by the Ministry of Health (http:/idengue.arsm.gov.my).
Description of data collection	Respondents were scientists and the public. The scientists included academicians, graduate students, research and scientific officers in environmental sciences, biological sciences, health and genetic sciences from universities and health-related governmental research institutes who were either involved in research or the control and prevention of dengue. The public respondents were sampled randomly from the population in dengue hotspots areas in the Klang Valley region, Malaysia. The survey was carried out face to face by trained enumerators with a bioscience background.
Data source location	Klang Valley, Malaysia.
Data accessibility	https://data.mendeley.com/datasets/4ky5krhf37/1

## Value of the Data

• Our data are important for assessing the social acceptance of Wolbachia-infected Aedes mosquitoes.
• Our data provide insight into understanding the intricate relationship between perceived benefits and risks with attitude towards Wolbachia-infected Aedes mosquitoes and the role of trust in key players, attitude towards nature versus material, attitude towards technology, and level of religiosity on perceived benefits and risks and attitude towards Wolbachia-infected Aedes mosquitoes.
• Our data can be useful and beneficial for governments, policymakers and institutions involved in environmental health and dengue control, to improve existing strategies to eradicate dengue as well as to improve existing policy to control dengue using multidisciplinary approaches.
• Our data can be used for further research and analysis e.g. to compare attitude and predict variables across demographic variables as well as structural equation modelling. A comparison with similar data from other countries can also be carried out.

## 1 Data Description

Dengue is a major environmental health issue that has affected economic growth and public health. Many vector control techniques have been introduced, including the Wolbachia-infected Aedes mosquitoes (WiAM) technique. The technique is to infect male Aedes mosquitoes with one of the bacteria known as Wolbachia [1]. Aedes mosquitoes with infected Wolbachia bacteria have the potential to help inhibit the transmission of dengue virus. Therefore, this study and dataset provide insightful information based on survey data on Malaysian stakeholders’ attitudes towards WiAM. The instrument was developed based on a previous study by Amin and Hashim (2015) on factors influencing attitudes towards the genetically modified Aedes mosquito (GMM) technique [2]. It includes six independent variables (perceived benefits, perceived risks, trust in key players, attitude towards nature versus material, attitude towards technology and religiosity), and uses attitudes towards Wolbachia-infected Aedes mosquitoes as the dependent variable. The content of the instrument was validated by seven researchers involved in the field of environmental health, measurement, consumer behaviour, and sustainable development. The questionnaires were prepared in Malay, translated into English, and certified by two translation experts. The administration of the survey was carried out face to face by trained graduate enumerators with a background in bioscience.

Referring to Table 1, 399 Malaysian stakeholders aged above 18 years old were surveyed from September 2016 to September 2017 to assess attitudes towards WiAM. The respondents comprised 204 females and 195 males; 50.6% were members of the public, and 55.6% were non-government employees. About 45.4% of the respondents were Muslim and 42.4% were Malay, which reflects the actual population ratio in the Klang Valley, where a majority are Malays and Muslims [3]. Additionally, more than 70% had a degree and above. This is due to half of the respondents being scientists while the remainder were from the general public. There is a high number of degree holders in the Klang Valley region due to the presence of three public universities in the area. Table 1 shows the demographic profiles of the respondents.

**Table 1:** Demographic profiles of respondents (*n* = 399).

Items		Frequency	Percentage %
Gender	Male	195	48.9
	Female	204	51.1
Stakeholders	Scientists	197	49.4
	Public	202	50.6
Sector of Occupations	Government	177	44.4
	Non-Government	222	55.6
Religion	Islam	181	45.4
	Other religions	218	54.6
Race	Malay	169	42.4
	Chinese	108	27.1
	Indian	91	22.8
	Others	31	7.8
Age (years old)	18-28	185	46.4
	29-39	132	33.1
	Above 40	78	19.5
Level of Education	High School and Pre-University	44	11.0
	Diploma	56	14.0
	Degree	163	40.9
	Masters and PhD	136	34.1

## 2 Social Acceptance of the WiAM Technique

### 2.1 Mean score and correlation analysis

**Fig. 1:**
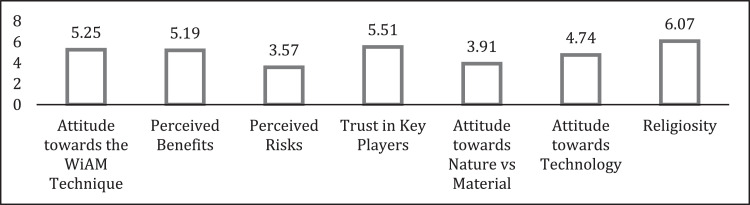
Mean Score of Attitude towards WiAM Technique.

Note: 1–2.99: Low; 3.00–5.00: Moderate; 5.01–7.00: High

As can be seen in Fig. 1, the stakeholders in Malaysia show a positive attitude towards WiAM (mean score = 5.25). It can also be seen that the perceived benefits of this technique (mean score = 5.19) were rated as high, while the risks were perceived as moderate (mean score = 3.57). Additionally, they professed a high level of religiosity (mean score = 6.07) and high trust in key players (mean score = 5.51). On the other hand, the mean score for attitude towards nature versus materials was about the mid-point value of 4.0 (mean score = 3.91), indicating that the respondents appreciated the importance of both nature and materials. Furthermore, they also have a moderately positive attitude towards technology (mean score = 4.74).

Subsequently, Pearson correlation analysis was conducted as suggested by Cheung and Chan (2005) [4] to determine the relationship between the variables. Table 2 shows a positive correlation between perceived benefits and attitude towards WiAM (*r* = 0.557, *p* = 0.000), and a negative relationship between perceived risks and attitude towards this technique (*r* = −0.278, *p* = 0.000). Trust in key players also highlighted a positive correlation with the attitude towards WiAM (*r* = 0.300, *p* = 0.000). At the same time, attitude towards technology also showed a significant association with the attitude towards WiAM (*r* = 0.276, *p* = 0.000), while religiosity showed a positive correlation with the attitude towards this technique (*r* = 0.198, *p* = 0.000). On the other hand, attitude towards nature versus material did not have any significant relationship with the attitude towards WiAM.

**Table 2:** The correlation matrix among the factors with attitude towards the WiAM technique.

		ATWT	PF	PR	TKP	NAT	TECH	REG
ATWT	Pearson Correlation	1	0.557^⁎⁎^	-0.278^⁎⁎^	0.300^⁎⁎^	-0.002	0.276**	0.198^⁎⁎^
	Sig. (2-tailed)		0.000	0.000	0.000	0.969	0.000	0.000
	N	399	399	399	399	399	3.99	399

Note: Attitude towards the WiAM Technique (ATWT), Perceived Benefits (PB), Perceived Risks (PR), Trust in Key Players (TKP), Attitude towards Nature versus Material (NAT), Attitude towards Technology (TECH), Religiosiy (REG); *p < 0.05, **p < 0.01 (2-tailed)

### 2.2 Multiple regression analysis

Multiple regression analysis (stepwise) was conducted to determine the factors contributing to attitude towards the Wolbachia-infected Aedes mosquitoes (see Table 3). Perceived risks, attitude towards nature versus material and religiosity did not significantly contribute to the change in attitude towards this technique. However, perceived benefits, attitudes towards technology, and trust in key players were found to contribute significantly to attitude towards WiAM. The findings from this analysis found that the most important predictor of attitude towards WiAM was perceived benefits (β = 0.488, *t* = 10.693, *p* = 0.000). This finding indicated that when the stakeholders perceived more benefits related to WiAM, then they will have a more positive attitude towards this technique. Frewer (2017) emphasized that when a person perceives that benefits are higher than risks, then they tend to support their choices [5].

**Table 3:** Multiple regression analysis.

Model summary				
Model	R	R Square	Adjusted R Square	Std. Error of the Estimate
1: (Constant), Perceived Benefits	0.557	0.310	0.309	0.868
2: (Constant), Perceived Benefits, Attitude towards Technology	0.91	0.326	0.322	0.859
3: (Constant), Perceived Benefits, Attitude towards Technology, Trust in Key Players	0.103	0.333	0.328	0.855

Coefficients				
Factors	B	Beta (β)	*T*	Significant Value
Perceived Benefits	0.483	0.488	10.693	0.000
Attitude towards Technology	0.91	0.121	2.816	0.005
Trust in Key Players	0.103	0.094	2.102	0.036
Attitude towards the WiAM technique (Constant)	1.721		5.869	0.009*

*F* value=65.792, *p* = 0.000 (*p* < 0.05)

⁎ Predictors: (Constant), Perceived Benefits, Attitude towards Technology, Trust in Key Players

The second significant predictor of attitude towards WiAM was the attitude towards technology (β = 0.121, *t* = 2.816, *p* = 0.005). This finding indicated that if they have a more positive attitude towards technology in general, then they will have a positive attitude towards WiAM. The third significant predictor was the factor of trust in key players (β = 0.094, *t* = 2.102, *p* = 0.036). The result showed that when the stakeholders have more trust in the key players, this enhances their positive attitude towards WiAM. The finding of this study was supported by Amin and Hashim's (2015) study which showed that the most significant contributing factor of attitude towards the GMM technique was perceived benefits [2]. Trust in key players and attitude toward technology were also significant predictors of attitude towards the GMM technique [2].

## 3 Experimental Design, Materials and Methods

In this data statement, a survey design was adopted. A total of 399 respondents replied from an initial target of 415. Respondents were selected randomly among stakeholders, including groups of scientists (academicians, researchers, and government officers) involved in dengue control, and public groups living in outbreak areas identified as Aedes hotspots in the Klang Valley according to statistics issued by the Ministry of Health, Malaysia. A multi-dimensional survey instrument measuring stakeholder attitudes towards WiAM was developed based on previously published research [2]. There were two sections of the questionnaire: section A consisted of questions reflecting on attitude towards WiAM and its potential predictors, while section B covered items of demographic background. All items were measured using a 7-point Likert scale, ranging from 1 (strongly disagree) to 7 (strongly agree). The Statistical Package for Social Sciences (SPSS®) software was used to evaluate descriptive and inferential statistics including the analysis of Pearson Correlation and Multiple Regression.

## Ethics Statement

No ethical approval was required in this study in compliance with the Guidelines for the Ethical Review of Clinical Research or Human Subject Study [6]. The participation of the respondents was entirely voluntary, and withdrawals were allowed at any time. The informed consent was collected from all respondents before the survey, and the respondent's details are kept confidential.

## Declaration of Competing Interest

The authors declare that they have no known competing financial interests or personal relationships that could have appeared to influence the work reported in this paper.

## Appendix Supplementary materials

Image, application 1

Image, application 2

Image, application 3
