# Supplementary material for: Perceived benefits and risks: A survey data set towards Wolbachia-infected Aedes Mosquitoes in Klang Valley
Source: Data Brief. 2020 Sep 2;32:106262. doi: 10.1016/j.dib.2020.106262 (PMC7481806; doi:10.1016/j.dib.2020.106262)
Supplement: Supplementary file 2 [file mmc2.docx]

***
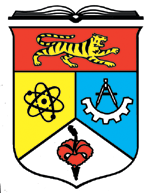
* UNIVERSITI KEBANGSAAN MALAYSIA**

**43600 UKM, BANGI**

**SELANGOR DARUL EHSAN**

**SOAL SELIDIK KAEDAH PENCEGAHAN DAN PENGAWALAN DENGGI DI MALAYSIA**

Dengan ini dimaklumkan bahawa Universiti Kebangsaan Malaysia sedang menjalankan satu kaji selidik berkenaan sejauh mana tahap kesedaran dan pandangan masyarakat Malaysia terhadap beberapa teknik yang digunakan untuk mencegah dan mengawal denggi, termasuklah kemungkinan pembebasan nyamuk Aedes yang diubahsuai secara genetik ke persekitaran. Kami amat menghargai sumbangan anda dalam membantu kami melengkapkan borang soal selidik yang dilampirkan ini. Semua maklumat yang diperoleh adalah semata-mata untuk tujuan penyelidikan dan akan dirahsiakan.

Sekian, terima kasih atas kerjasama yang diberi.

Prof. Dr. Latifah Amin (Ketua Projek)

**SOAL SELIDIK TEKNIK PENCEGAHAN DAN PENGAWALAN DENGGI DI MALAYSIA**

| **PANDUAN:** Sila bulatkan nombor yang berpadanan dengan jawapan anda. Semua maklumat peribadi anda akan dirahsiakan.  **Contoh:** Adakah bioteknologi moden berguna kepada masyarakat?   \| Sangat Sangat  tidak setuju  setuju \| \| --- \| \| **1 2 3 4 5 6 7** \| |
| --- | --- | --- |

**SEKSYEN A**:

Sila bulatkan nombor yang berpadanan dengan jawapan anda.

**PERSEPSI FAEDAH**

| Bakteria *Wolbachia* yang berupaya memendekkan jangka hayat nyamuk Aedes. | Sangat Sangat  tidak setuju  setuju |
| --- | --- |
| S1. Kaedah berikut meningkatkan mutu hidup masyarakat Malaysia. | **1 2 3 4 5 6 7** |
| S2. Kaedah berikut berfaedah kepada masyarakat di Malaysia. | **1 2 3 4 5 6 7** |
| S3. Kaedah berikut berfaedah dalam mencegah wabak demam denggi. | **1 2 3 4 5 6 7** |
| S4. Kaedah berikut berkesan membasmi denggi. | **1 2 3 4 5 6 7** |
| S5. Kaedah berikut berfaedah kepada kesihatan saya dan keluarga. | **1 2 3 4 5 6 7** |
| S6. Kaedah berikut lebih bermanfaat kepada masyarakat berbanding risiko. | **1 2 3 4 5 6 7** |
| S7. Apa sahaja risiko disebabkan kaedah berikut boleh diatasi dengan kajian pada masa akan datang. | **1 2 3 4 5 6 7** |

**PERSEPSI RISIKO**

| Bakteria *Wolbachia* yang berupaya memendekkan jangka hayat nyamuk Aedes. | Sangat Sangat  tidak setuju  setuju |
| --- | --- |
| S1. Tahap kerisauan anda mengenai kesan yang tidak diketahui melalui kaedah berikut. | **1 2 3 4 5 6 7** |
| S2. Kesan buruk akibat kaedah berikut akan muncul selepas jangka masa panjang. | **1 2 3 4 5 6 7** |
| S3. Kaedah berikut memudaratkan generasi akan datang. | **1 2 3 4 5 6 7** |
| S4. Kaedah berikut berkemungkinan mengundang kesan baru yang belum diketahui. | **1 2 3 4 5 6 7** |
| S5. Kaedah berikut boleh mengakibatkan malapetaka yang besar kepada masyarakat Malaysia. | **1 2 3 4 5 6 7** |
| S6. Tahap kerisauan tentang kemungkinan bahaya kepada kesihatan anda dan keluarga. | **1 2 3 4 5 6 7** |
| S7. Kesan kaedah berikut sangat berbahaya. | **1 2 3 4 5 6 7** |

**SIKAP TERHADAP TEKNIK WIAM**

| Bakteria *Wolbachia* yang berupaya memendekkan jangka hayat nyamuk Aedes. | Sangat Sangat  tidak setuju  setuju |
| --- | --- |
| S1. Kaedah berikut patut dilakukan secara besar-besaran dan dikomersialkan. | **1 2 3 4 5 6 7** |
| S2. Kerajaan patut memberi lebih banyak bantuan kewangan untuk membangunkan kaedah berikut. | **1 2 3 4 5 6 7** |
| S3. Kaedah berikut membantu dalam mengurangkan kadar kematian dikalangan  komuniti. | **1 2 3 4 5 6 7** |
| S4. Kaedah berikut adalah perlu untuk membasmi denggi. | **1 2 3 4 5 6 7** |
| S5. Kaedah berikut patut digalakkan. | **1 2 3 4 5 6 7** |

**KEYAKINAN TERHADAP PIHAK BERKEUTAMAAN**

| Pihak berikut menjalankan tugas kerja dengan baik untuk masyarakat. | Sangat Sangat  tidak setuju  setuju |
| --- | --- |
| S1. Ahli Sains/Penyelidik Universiti dan Institusi Penyelidikan. | **1 2 3 4 5 6 7** |
| S2. Industri Pengeluaran Racun Serangga dan Farmaseutikal. | **1 2 3 4 5 6 7** |
| S3. Jabatan kerajaan yang terlibat dalam pengawalan seperti Kementerian Kesihatan Malaysia (KKM) dan Jabatan Biokeselamatan. | **1 2 3 4 5 6 7** |

**SIKAP TERHADAP ALAM SEMULAJADI vs MATERIAL**

| Perspektif A | Cenderung Cenderung  ke A ke B  **1 2 3 4 5 6 7** | Perspektif B |
| --- | --- | --- |
| S1a) Masyarakat yang memelihara alam semula jadi pada keadaan asalnya. | **1 2 3 4 5 6 7** | S1b) Masyarakat yang menekankan penggunaan alam semula jadi untuk mendapat kemewahan. |
| S2a) Masyarakat yang mengamalkan sistem ekonomi perancangan berpusat. | **1 2 3 4 5 6 7** | S2b) Masyarakat yang bergantung kepada ekonomi yang berdasarkan system pasaran |
| S3a) Masyarakat yang akan menyekat kemajuan sekiranya terdapat sebarang bahaya/risiko. | **1 2 3 4 5 6 7** | S3b) Masyarakat yang sanggup menerima sebarang bahaya untuk mencapai kemewahan |
| S4a) Masyarakat yang mengutamakan pemeliharaan alam sekitar berbanding perkembangan ekonomi. | **1 2 3 4 5 6 7** | S4b) Masyarakat yang mengutamakan perkembangan ekonomi berbanding pemeliharaan alam sekitar. |
| S5a) Malaysia yang menekankan bahawa alam semula jadi adalah rapuh dan mudah dirosakkan manusia. | **1 2 3 4 5 6 7** | S5b) Masyarakat yang menekankan bahawa alam semula jadi boleh menahan tindakan manusia. |

**SIKAP TERHADAP TEKNOLOGI**

|  | Sangat tidak Sangat  setuju setuju |
| --- | --- |
| S1. Teknologi moden telah membuatkan manusia hilang hormat keatas alam semula jadi. | **1 2 3 4 5 6 7** |
| S2. Pembangunan sains dan teknologi membuatkan manusia focus terhadap keuntungan berbanding pembangunan alam semula jadi. | **1 2 3 4 5 6 7** |
| S3. Kebergantungan manusia kepada teknologi moden telah memusnahkan nilai kemanusiaan. | **1 2 3 4 5 6 7** |
| S4. Perkembangan sains dan teknologi yang tiada had, akhirnya akan menuju kepada kemusnahan perikemanusiaan. | **1 2 3 4 5 6 7** |
| S5. Teknologi moden telah menggugat keseimbangan alam semula jadi | **1 2 3 4 5 6 7** |
| S6. Kewujudan industry dan teknologi terhadap kehidupan Bandar telah menimbulkan masalah dan kerunsingan. | **1 2 3 4 5 6 7** |

**KOMITMEN KEAGAMAAN**

|  | Sangat tidak Sangat  setuju setuju |
| --- | --- |
| S1. Agama adalah penting dalam kehidupan saya. | **1 2 3 4 5 6 7** |
| S2. Pandangan agama adalah penting apabila saya perlu membuat keputusan mengenai isu-isu  kontroversi. | **1 2 3 4 5 6 7** |
| S3. Bersembahyang adalah penting dalam hidup saya. | **1 2 3 4 5 6 7** |
| S4. Membaca kitab agama adalah penting dalam hidup saya. | **1 2 3 4 5 6 7** |
| S5. Agama adalah sangat penting kepada saya kerana ia menjawab banyak persoalan tentang erti  hidup. | **1 2 3 4 5 6 7** |
| S6. Agama menawarkan saya ketenangan apabila dilanda kesedihan dan musibah. | **1 2 3 4 5 6 7** |
| S7. Saya berusaha untuk hidup berlandaskan kepercayaan agama saya. | **1 2 3 4 5 6 7** |
| S8. Tiada apa yang boleh belaku tanpa keizinan Tuhan. | **1 2 3 4 5 6 7** |

**SEKSYEN B:**

Soalan-soalan berikut hanyalah untuk analisis umum. Maklumat peribadi anda akan dirahsiakan.

S1. **Jantina**: 1. Lelaki 2. Perempuan

S2. **Pihak yang berkepentingan**: 1. Saintis 2. Orang Awam

S3. **Sektor Pekerjaan**: 1. Kerajaan 2. Bukan Kerajaan

S4. **Agama**: 1. Islam 2. Lain-lain Agama

S5. **Bangsa**: 1. Melayu 2. Cina 3. India 4. Lain-lain

S6. **Umur**: 1. 18-28 tahun 2. 29-39 tahun 3. 40 tahun ke atas

S7. **Tahap pendidikan**: 1. Menengah dan Pra Universiti 2. Diploma 3. Ijazah 4. Sarjana dan PhD

**TERIMA KASIH**
